# Corncob Returning Enhances Soil Fertility and Rhizosphere Microbiome Functions to Improve Growth and Nutrient Uptake of *Eleutherococcus sessiliflorus* in Cold Agroecosystems

**DOI:** 10.3390/biology14121735

**Published:** 2025-12-04

**Authors:** Qian Liu, Ying Qu, Shan Jiang, Xingchi Guo, Yuhe Xing, Junyan Zheng, Zhiyu Dong, Wei Yu, Guoyu Zhang

**Affiliations:** 1 College of Landscape Architecture, Changchun University, Changchun 130012, China; 2 Institute of Resource Utilization and Soil Conservation, Changchun University, Changchun 130022, China

**Keywords:** corncob returning, *Eleutherococcus sessiliflorus*, soil microbial biomass, enzyme activity, soil fertility, rhizosphere processes, PLS-SEM, sustainable agriculture, circular bioeconomy, cold-region farming

## Abstract

Corncob residues, an abundant but underutilized organic resource in Northeast Asia, offer substantial potential for improving soil health and plant productivity. This study investigates the effects of corncob returning on soil physicochemical properties, microbial processes, and the performance of *Eleutherococcus sessiliflorus* in a cold–temperate region (Jilin Province, China). The treatments included no-amendment control (CK), corncob incorporation (CI), and corncob mulching (CM). Corncob returning significantly increased soil organic carbon, moisture content, and the availability of N–P–K, while reducing soil bulk density, thus improving soil structure and nutrient availability. Both CI and CM treatments enhanced microbial biomass C, N, and P, as well as nutrient-cycling enzyme activities (β-glucosidase, urease, and alkaline phosphatase), accelerating C–N–P turnover in the rhizosphere. These improvements resulted in enhanced plant nutrient status and significant gains in biomass, with plant height and fruit number increasing by up to 44% and 136%, respectively. Multivariate analysis and PLS-SEM revealed that soil improvements strongly stimulated enzyme activity (path coefficient = 0.956), and enhances the microbial niche, thereby promoting plant traits through nutrient release (enzyme → plant path coefficient = 0.694). Microbial functional activity, rather than microbial richness, plays a more crucial role in plant growth promotion. Collectively, these findings underscore that corncob returning improves *E. sessiliflorus* performance through a soil biochemical activation pathway mediated by microbial metabolism and enzymatic nutrient release. This study provides strong evidence supporting corncob recycling as a cost-effective, environmentally sustainable approach for improving medicinal plant production and advancing circular agriculture in cold-region ecosystems.

## Simple Summary

Agricultural residues are often wasted or burned, leading to resource loss and environmental pollution. Corncobs are a readily available by-product in Northeast China, yet their potential value in ecological cultivation systems remains under-explored. In this study, we returned corn residues to the soil of *Eleutherococcus sessiliflorus*, a valuable medicinal and edible plant, to evaluate their effects on soil health and plant growth. We found that corncob application improved soil nutrients, increased beneficial soil microorganisms, and enhanced enzyme activities that help release nutrients. As a result, plants grew faster, had larger leaves and thicker stems, and produced more fruit. Our findings show that corncob recycling not only promotes sustainable agriculture but also provides a low-cost and eco-friendly solution for waste management, particularly in cold region farming systems. This research offers a practical pathway to improve medicinal plant production while advancing circular agriculture and resource reuse.

## 1. Introduction

The transition from conventional fertilization toward circular and low-carbon agriculture has accelerated the search for sustainable nutrient sources and soil restoration strategies [1]. Recycling crop residues as soil amendments is increasingly recognized as an effective approach to improve soil quality, stimulate microbial processes, and reduce environmental burdens associated with agricultural waste disposal [2]. Northeast China, one of the major maize-producing regions globally, generates substantial corncob biomass each year. Although rich in lignocellulosic carbon and possessing high porosity, corncob residues remain underutilized, often burned or discarded, resulting in nutrient loss and air pollution concerns [3]. Returning corncob to farmland offers an opportunity to enhance soil structure, increase organic matter input, and support microbial-mediated nutrient turnover, especially in soils with low organic inputs and degraded biological activity [4].

The rhizosphere acts as a dynamic interface where plant roots, soil, and microorganisms interact to regulate nutrient availability, enzyme activities, and plant health [5]. Organic residue inputs can reshape rhizosphere microbial networks by supplying carbon substrates, altering soil microhabitats, and stimulating beneficial decomposers and nutrient-cycling taxa [6]. However, the outcomes of residue application are strongly influenced by management practices. Incorporation into deeper soil layers enhances residue–microbe contact and decomposition rates, whereas surface mulching may improve moisture retention, temperature buffering, and root zone stability [7]. Understanding the relative performance of these strategies is particularly important in cold regions, where low temperatures slow organic matter turnover and restrict root development.

*Eleutherococcus sessiliflorus*, a perennial medicinal shrub widely used in traditional medicine and functional health industries, is an economically important species in Northeast Asia. Despite its ecological adaptability, *E. sessiliflorus* cultivation often suffers from slow nutrient accumulation, limited root vigor, and soil fertility decline under long-term cultivation. Enhancing rhizosphere function through sustainable residue management may help overcome these constraints, yet research on organic residue returning in medicinal herb systems remains scarce. Moreover, little information is available on how corncob amendments affect soil biochemical properties, rhizosphere microbiome assembly, and nutrient acquisition in perennial medicinal crops.

To address this knowledge gap, we conducted a multi-season field experiment to compare two corncob returning strategies—deep incorporation and surface mulching—with a no-amendment control in *E. sessiliflorus* production systems. We hypothesized that (i) corncob application would improve soil physicochemical properties and enzyme activities; (ii) rhizosphere microbial diversity, community composition, and nutrient-cycling functions would be enhanced; (iii) these improvements would promote nutrient uptake and biomass accumulation in *E. sessiliflorus*, with mulching expected to provide greater benefits in cold-region environments. To address the proposed hypotheses, the specific objectives of this study are defined as follows: (i) To quantify the effects of corncob incorporation (CI) and mulching (CM) on key soil physicochemical properties and enzyme activities in the rhizosphere of *E. sessiliflorus*. (ii) To characterize shifts in rhizosphere microbial diversity, community composition, and functional potential (nutrient cycling-related taxa) induced by corncob returning treatments. (iii) To evaluate the impacts of soil and microbial changes on nutrient uptake, growth traits (plant height, biomass), and reproductive performance (fruit number) of *E. sessiliflorus*, and to compare the effectiveness of CI and CM in cold-temperate agroecosystems. Findings from this study provide new insights into residue-based soil improvement strategies for medicinal crop cultivation and contribute to advancing bio-circular agriculture in temperate ecosystems.

## 2. Materials and Methods

### 2.1. Study Site and Experimental Design

The field experiment was conducted from September 2022 to October 2024 at the experimental teaching base of Changchun University, Chaoyang District, Changchun City, Jilin Province, China (43°49′50″ N, 125°17′58″ E). The region has a typical temperate monsoon climate with a mean annual precipitation of 860.3 mm, average annual temperature of 7.3 °C, mean sunshine duration of 2184 h, and annual solar radiation of 1563.4 kWh/m^2^.

**Figure 1:**
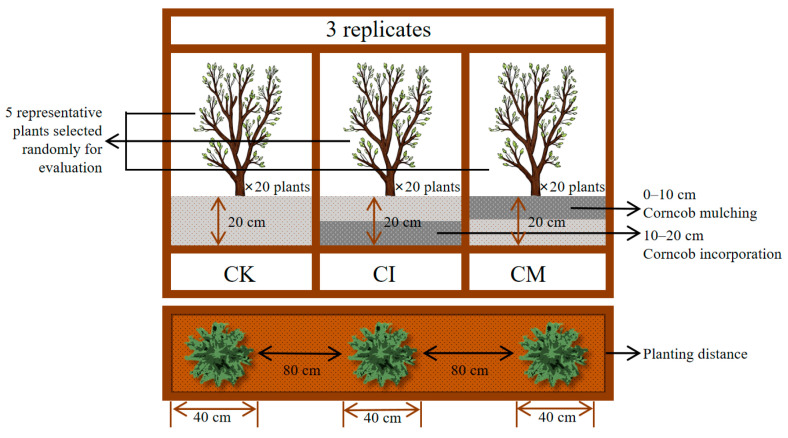
Experimental design diagram: A randomized block design with three treatments and three replicates, with 20 *E*. *sessiliflorus* plants planted per plot.

The field was planted with *E. sessiliflorus* seedlings in October 2022, and a randomized block design was used with three treatments and three replicates, each plot measuring 30 m^2^ (6 m × 5 m), with 20 *Eleutherococcus sessiliflorus* plants planted per plot. For plant assessment, five representative plants were randomly selected from each plot for growth evaluation. The treatments included the following: CK (no amendment, control), CI (corncob incorporation at a 20 cm depth), and CM (corncob mulching, lightly mixed into the 0–10 cm surface layer). The corncob biomass used in the study was sourced from Zea mays (cv. Jingqing 707) residues collected from Fuyu, Jilin Province. The corncobs were air-dried, crushed to ≤5 mm, and applied at a rate of 2.5 t C/ha, equivalent to 16.7 kg per plot. A polyethylene (PE) film root barrier (50 cm wide) was installed between plots to minimize belowground interference. Field management followed local agronomic practices without the use of chemical fertilizers (Figure 1). This study did not include co-cultivation experiments with Fabaceae species. Although leguminous plants are known for their nitrogen-fixing ability, which could potentially enhance nutrient availability and amplify the effects of corncob amendment, the primary aim of this study was to evaluate the effects of corncob return on the growth of *E. sessiliflorus* alone. Future studies could consider co-cultivation with leguminous species to further assess the impact of corncob return on nutrient supply and growth across different plant types.

### 2.2. Soil and Plant Sampling

Soil and plant sampling was conducted during fruit maturity in October 2024. Soil was collected from the 0–20 cm layer using an S-shaped sampling method, with each composite sample consisting of five cores per plot after removal of surface residues. The fresh soil was sieved through a 2 mm mesh, divided into subsamples, and processed for different analyses: fresh soil was stored at −80 °C for microbial and enzyme analyses, while air-dried soil was used for physicochemical analysis. Roots and litter fragments were manually removed during processing. For plant assessment, five representative *E. sessiliflorus* plants were harvested from each plot, and biomass, nutrient content, and root characteristics were measured immediately after harvest.

### 2.3. Soil Physicochemical Analysis

Soil pH was measured in a 1:2.5 soil-to-water suspension using a digital pH meter [8]. Gravimetric soil moisture content was determined via oven-drying at 105 °C [9]. Bulk density was measured using the core method, and porosity was calculated accordingly [10]. Available nitrogen (AN) was quantified by alkaline hydrolysis diffusion, available phosphorus (AP) by molybdenum–antimony colorimetry, and available potassium (AK) by flame photometry [11]. Soil organic carbon (SOC) was determined by dichromate oxidation–ferrous sulfate titration [12]. Microbial biomass carbon (MBC), nitrogen (MBN), and phosphorus (MBP) were analyzed via chloroform fumigation–extraction [13] to assess microbial nutrient pools.

### 2.4. Soil Enzyme Activities

Key rhizosphere enzyme activities were determined to evaluate nutrient cycling functions. Urease activity was measured using colorimetric detection of ammonium released from urea hydrolysis [14]. Sucrase activity was assayed via 3,5-dinitrosalicylic acid colorimetry after sucrose incubation [15]. Soil alkaline phosphates were determined by p-nitrophenyl phosphate hydrolysis [16]. Enzyme activity was expressed per gram of dry soil.

### 2.5. Microbial Community Analysis

DNA was extracted from 0.5 g fresh soil using a commercial soil DNA isolation kit (Magentec, Guangzhou, China; catalog no. D3142-02). Bacterial 16S rRNA genes (V3–V4 region) and fungal ITS regions were amplified using universal primers [17]. Amplicons were sequenced on an Illumina platform following manufacturer protocols. Raw reads were processed using a standard pipeline: quality filtering, merging, chimera removal, and clustering into OTUs/ASVs at 97% similarity. Taxonomic annotation referenced the SILVA and UNITE databases [18]. α-diversity indices (Shannon, Chao1, Simpson, ACE) and β-diversity (PCoA/Bray–Curtis) were computed using QIIME or equivalent tools.

### 2.6. Plant Growth and Nutrient Measurement

Plant growth parameters, including height, stem diameter, and leaf number, were recorded monthly for 5 plants per replicate. At harvest, shoot and root biomass were measured for 10 plants per treatment and replicate, and were oven-dried at 65 °C to constant weight. Leaf N, P, and K concentrations were determined using Kjeldahl digestion, molybdate colorimetry, and flame photometry, respectively [19].

### 2.7. Statistical Analysis

Data were analyzed using R 4.4.2 software. One-way ANOVA followed by Tukey’s HSD test assessed treatment effects. α-diversity indices, ordination analyses, and heatmaps were generated with vegan and phyloseq packages. Community differences were visualized via PCoA. Correlations were tested using Pearson analysis. Differences were considered significant at *p* < 0.05. Graphs were plotted using ggplot2. Prior to statistical analysis, the normality of data distribution was tested using the Shapiro–Wilk test, and the homogeneity of variance was verified via Levene’s test. For datasets that did not initially meet normality assumptions, a log-transformation (log_10_(x + 1)) was applied to normalize the distribution, ensuring compliance with the requirements of parametric tests (one-way ANOVA). For Pearson correlation analysis, the strength of correlations was defined based on conventional thresholds: low correlation (|r| = 0.1–0.3), medium correlation (|r| = 0.3–0.5), and high correlation (|r| ≥ 0.5). This classification facilitated clear interpretation of the magnitude of linear relationships between soil properties, microbial indicators, and plant traits. This study focuses on the effects of a single categorical independent variable (corncob returning treatments: CK, CI, CM) on multiple dependent variables (e.g., soil nutrients, enzyme activities, plant growth traits). The research objectives were achieved through two complementary methods: (i) One-way ANOVA (coupled with Tukey’s HSD post hoc test) was used to evaluate the effect of each treatment on individual dependent variables, ensuring accurate detection of differences among various indicators. (ii) Redundancy Analysis (RDA) and Partial Least Squares Structural Equation Modeling (PLS-SEM) were employed to explore the multivariate relationships between the independent variable group and dependent variable groups. Compared with MANOVA, these methods more effectively meet the research needs: they not only quantify the treatment effects but also clarify the mechanistic associations among soil-microbial-plant variables, which is the core focus of this study.

## 3. Results and Discussion

### 3.1. Effects of Corncob Returning on Soil Physicochemical Properties

#### 3.1.1. Soil pH and Electrical Conductivity

Corncob returning treatments exerted a moderate yet distinct effect on soil pH (Table 1). In the 0–10 cm layer, CI (8.35) and CM (8.12) slightly reduced soil alkalinity compared with CK (8.59), with mulching (CM) showing the greatest decline. A similar trend was observed in the 10–20 cm layer, where CM reduced pH to 8.59 relative to 9.24 in CK. The decrease in pH likely reflects partial decomposition of corncob material and generation of organic acids during microbial metabolism [20,21]. This finding is consistent with studies reporting that incorporation of high-carbon residues and lignocellulosic biomass can buffer alkaline soils and promote proton release through microbial respiration and organic acid secretion [22]. Electrical conductivity (EC) exhibited a slight increase under CI at the surface layer (0.19 mS/cm) relative to CK (0.11 mS/cm), whereas CM had comparatively modest effects. This suggests that incorporation enhanced mineralization and release of soluble ions, whereas mulching slowed ionic release due to gradual decomposition on the soil surface [23]. However, all EC values remained low (<0.20 mS/cm), indicating no risk of soil salinization under corncob application. The low EC values and absence of salinization risk under corncob returning can be attributed to three interrelated mechanisms. First, corncob residues are inherently low in soluble salts (electrolytes), with elemental analysis showing that the content of soluble Na^+^, Cl^−^, and SO_4_^2−^ in the applied corncob biomass was <0.1 g/kg dry weight—far below the threshold for inducing salt accumulation in soil. Second, the porous fibrous structure of corncobs enhances soil water infiltration and hydraulic conductivity, facilitating leaching of minor soluble ions (e.g., K^+^ released from corncob decomposition) downward to deeper soil layers (10–20 cm), as evidenced by the slightly higher EC in the 10–20 cm layer of CI plots (0.14 mS/cm) compared to the surface (0.19 mS/cm; Table 1). This leaching effect prevents the buildup of soluble ions in the root zone (0–10 cm), where EC is most relevant to plant salt stress. Third, the stimulation of microbial biomass and activity (Section 3.3) promotes the immobilization of soluble cations (e.g., NH_4_^+^, K^+^) into microbial biomass, reducing the concentration of free electrolytes in the soil solution—a key driver of EC. Collectively, these mechanisms (low salt input, enhanced leaching, microbial immobilization) explain why corncob application does not pose a salinization risk, even with long-term residue addition.

#### 3.1.2. Soil Moisture and Bulk Density

Corncob application markedly improved soil moisture conditions. In the 0–10 cm layer, moisture increased from 14.98% in CK to 18.34% in CI and 25.37% in CM, with CM presenting the most pronounced enhancement (Table 1). This pattern was consistent at 10–20 cm depth. As corncob possesses a porous fibrous structure and high water-holding capacity, residue mulching can effectively reduce surface evaporation and enhance water retention in cold, semi-humid regions [24]. Such mulching-mediated moisture conservation has also been documented in cereal and orchard systems receiving organic residue inputs [25,26]. Bulk density (BD) decreased significantly under both CI and CM relative to CK, with CM consistently showing the lowest BD values (1.12 g/cm^3^ at 0–10 cm and 1.23 g/cm^3^ at 10–20 cm). These improvements likely result from enhanced soil aggregation, increased pore space, and gradual incorporation of labile organic matter by soil fauna and microbes [27]. Lower BD favors root penetration, aeration, and microbial activity, ultimately contributing to improved rhizosphere functioning.

#### 3.1.3. Soil Organic Carbon

Both CI and CM significantly increased soil organic carbon (SOC) compared with CK. At 0–10 cm, SOC increased from 16.25 g/kg (CK) to 18.18 g/kg (CI) and 20.47 g/kg (CM), with similar trends at 10–20 cm. The greater SOC enhancement under mulching reflects slower decomposition and continuous carbon input at the surface, alongside reduced carbon loss via oxidation [28]. Returning lignocellulosic residues has been shown to stimulate humification and contribute to long-term soil carbon stabilization [29], consistent with the notable SOC gains observed in this experiment.

#### 3.1.4. Available Nutrients

**Figure 2:**
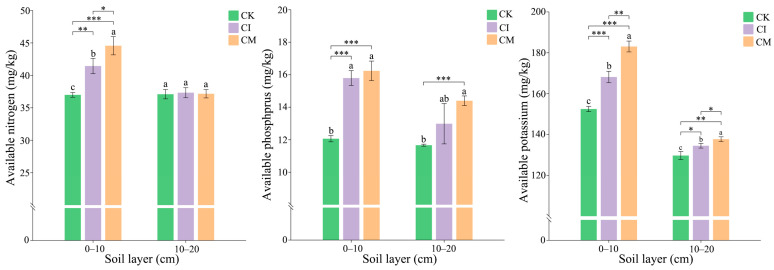
Effects of corncob returning on soil available nitrogen (AN), available phosphorus (AP), and available potassium (AK) in *E. sessiliflorus* rhizosphere at 0–10 cm and 10–20 cm soil depths. Significance levels: * *p* < 0.05, ** *p* < 0.01, *** *p* < 0.001. Different lowercase letters indicate significant differences among the CK, CI, and CM treatments at the same soil depth (*p* < 0.05), as determined by one-way analysis of variance (ANOVA) followed by Tukey’s honestly significant difference (HSD) post hoc test.

The results showed that corncob returning significantly improved soil nutrient availability, particularly available nitrogen (AN), phosphorus (AP), and potassium (AK), across both soil layers (0–10 cm and 10–20 cm) compared to the control (Figure 2). AN was significantly higher in both CI and CM treatments compared to CK, especially in the 0–10 cm layer. The increase in available N under CI and CM indicates enhanced nitrogen mineralization and microbial activity in the rhizosphere, driven by the decomposition of corncob residues. Nitrogen availability is crucial for plant growth, and the observed increase suggests that corncob residues can effectively contribute to soil nitrogen cycling by supporting nitrogen-fixing and decomposer microorganisms [30]. Similarly, AP showed significant increases under both CI and CM treatments, with CM generally showing the highest values in both soil layers. This enhancement reflects the greater phosphorus mobilization facilitated by organic amendments. The increased P availability in corncob-treated soils may result from microbial-mediated phosphorus solubilization, as well as improved soil aggregation and reduced phosphorus fixation due to corncob’s effect on soil structure and organic matter content [31]. AK followed a similar pattern, with CI and CM treatments significantly increasing K levels compared to CK, particularly in the 0–10 cm layer. The increase in available K in response to corncob returning may be attributed to the release of potassium from the corncob residues themselves, as well as enhanced microbial processes that increase potassium solubility and uptake [32]. The higher K content is also likely linked to improved soil moisture retention and microbial activity under the mulching treatment, which helps maintain potassium availability in the soil solution.

### 3.2. Effects of Corncob Returning on Soilbiological Properties

#### 3.2.1. Effects of Corncob Returning on Soil Microbial OTU Richness

**Figure 3:**
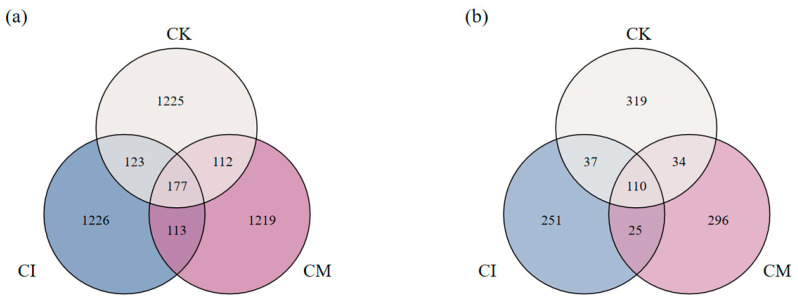
Venn diagrams of (**a**) bacterial and (**b**) fungal OTUs in rhizosphere soils under different corncob returning treatments.

Residue returning notably altered soil microbial richness, as shown by the Venn diagrams of bacterial and fungal OTUs (Figure 3). The bacterial community displayed the greatest richness under CI (1226 unique OTUs), followed by CM (1219), while CK harbored comparatively fewer unique taxa (1225). Moreover, the shared OTU number among all treatments (177) suggests the presence of a core bacterial consortium associated with *E. sessiliflorus* rhizosphere, regardless of residue management. The increase in unique bacterial OTUs under residue treatments indicates that corncob returning introduced new ecological niches and substrate heterogeneity, stimulating colonization by diverse taxa [33]. Lignocellulosic residues supply complex carbon sources that foster copiotrophic bacteria and support metabolic diversification [34], explaining the enrichment of distinct bacterial pools under CI and CM. The slightly higher richness under CI implies that incorporation enhances substrate–microbe contact, accelerating residue turnover and stimulating bacterial proliferation [35].

Fungal communities also responded strongly to corncob amendment. CM exhibited the highest number of unique fungal OTUs (296), exceeding CI (251) and CK (319). Meanwhile, 110 fungal OTUs were shared across all treatments, representing a stable rhizosphere core mycobiome in this system. The enhanced fungal diversity under CM suggests that surface mulching may offer more favorable microhabitat conditions, such as sustained moisture and residue–hypha contact, which facilitate fungal colonization and lignocellulose degradation [36]. Fungi are primary decomposers of recalcitrant plant residues, and mulching likely created a surface environment rich in polysaccharides and humic precursors, encouraging fungal recruitment and niche partitioning [37,38].

#### 3.2.2. Effects of Corncob Returning on Bacterial Community Composition

**Figure 4:**
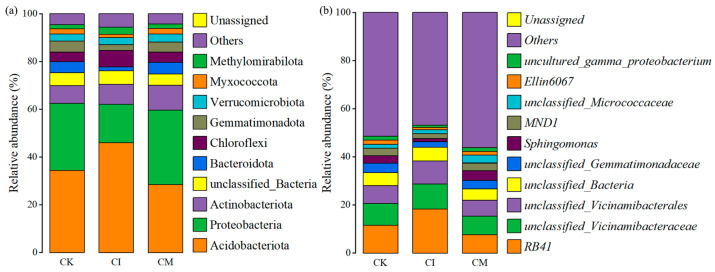
Relative abundance of dominant soil bacterial taxa at (**a**) phylum and (**b**) genus levels under different corncob returning treatments.

Corncob amendments reshaped the rhizosphere bacterial community structure at both the phylum and genus levels (Figure 4). Although Acidobacteriota, Proteobacteria, Actinobacteriota, and Bacteroidota dominated all treatments, their relative abundances varied under different residue management strategies.

Compared with CK, both CI and CM treatments increased the abundance of Proteobacteria and Bacteroidota, while reducing Acidobacteriota (Figure 4a). Proteobacteria are typically considered copiotrophic groups stimulated by readily available nutrients and labile carbon sources [39], suggesting that corncob returning enhanced nutrient supply and created resource-rich microhabitats. Bacteroidota are known decomposers of complex polysaccharides, particularly hemicellulose [40], implying active involvement in lignocellulose degradation following corncob addition. In contrast, the decline in Acidobacteriota—which generally thrives in low-nutrient or acidic environments—indicates a shift toward more nutrient-favorable microbial niches [41]. Additionally, increases in Gemmatimonadota and Chloroflexi under mulching suggest enhanced soil moisture and carbon turnover, as these groups benefit from carbon mineralization processes and improved microaerobic conditions [42]. Overall, CM demonstrated a more pronounced shift in phylum-level composition than CI, reflecting stronger stimulation of copiotrophic and residue-degrading taxa.

At the genus level, RB41, unclassified_Vicinamibacteraceae, unclassified_Vicinamibacterales, and taxa within Gemmatimonadaceae were dominant in all treatments, representing core rhizosphere bacteria in *E. sessiliflorus* soils (Figure 4b). Notably, CM enriched Sphingomonas, Ellin6067, and members of MND1, whereas CK showed relatively higher abundance of oligotrophic taxa. Sphingomonas is widely recognized for degrading aromatic and complex carbon compounds, promoting soil organic matter turnover, and supporting plant growth through phytohormone production and stress mitigation [43]. Enrichment of this genus under CM suggests enhanced decomposition activity and possible improvement in plant growth-promoting functions. Similarly, increased abundance of MND1 and Ellin6067, taxa associated with nitrogen transformation and carbon cycling, indicates active nutrient mobilization under residue mulching [44]. Conversely, the reduction in unclassified or oligotrophic bacteria in residue-treated soils suggests a transition toward metabolically active communities adapted to fresh organic input and enriched nutrient environments [45].

#### 3.2.3. Effects of Corncob Returning on Soil Bacterial α-Diversity

Corncob addition influenced the α-diversity of rhizosphere bacterial communities, as reflected by richness (ACE, Chao1) and diversity indices (Shannon, Simpson) (Table 2). Both corncob incorporation (CI) and corncob mulching (CM) tended to increase bacterial richness, although the magnitude varied among indices. The CM treatment exhibited the highest ACE (1305) and Chao1 (1329), significantly exceeding CK and slightly higher than CI. These results indicate that corncob returning promoted the establishment of a more diverse bacterial reservoir in the root zone.

Increases in richness indices under residue application suggest that lignocellulosic carbon inputs enhanced substrate heterogeneity and energy availability, supporting the proliferation of niche-differentiated bacterial taxa [46]. Previous studies have also reported that organic residue incorporation increases microbial richness by supplying carbon sources and modifying microhabitats through improved soil aggregation and moisture retention [47]. Despite differences in richness, Shannon and Simpson indices remained statistically comparable across treatments, indicating that community evenness and dominance patterns were relatively stable. This suggests that while corncob returning recruited additional bacterial taxa, it did not drastically disturb the core microbial structure or introduce strong competitive exclusion [48]. Such a balanced diversity response may reflect gradual carbon release during residue decomposition and the ability of rhizosphere bacterial communities to maintain functional stability under moderate organic amendments.

#### 3.2.4. Effects of Corncob Returning on Fungal Community Composition

**Figure 5:**
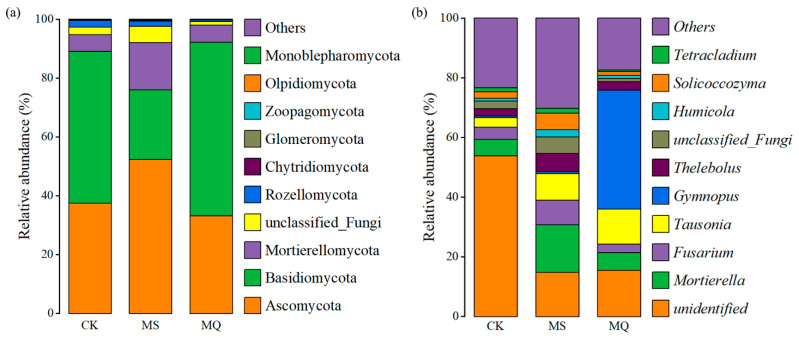
Relative abundance of major fungal taxa at (**a**) phylum and (**b**) genus levels under different corncob returning treatments.

Corncob returning substantially modified the soil fungal community at both phylum and genus levels (Figure 5). Ascomycota are frequently associated with oligotrophic environments and stress-adapted saprotrophs [49]. Their reduction suggests improved nutrient availability and reduces environmental stress following corncob addition. In contrast, Basidiomycota and Mortierellomycota play critical roles in lignocellulose decomposition and nutrient mineralization [50], and their enrichment indicates enhanced breakdown of corncob-derived organic matter. Notably, CM exhibited a stronger increase in Basidiomycota than CI, consistent with mulching promoting stable moisture and surface organic substrate, thereby favoring hyphal growth and lignin-degrading activity [51]. The presence of Glomeromycota across treatments reflects active arbuscular mycorrhizal associations in *E. sessiliflorus* rhizosphere, potentially contributing to improved nutrient mobilization under organic inputs [52].

At the genus level, Mortierella was highly enriched under CM compared to both CK and CI, highlighting the role of surface residue in stimulating saprotrophic fungi (Figure 5b). Mortierella spp. are widely recognized for promoting carbon turnover, producing polyunsaturated fatty acids, and enhancing phosphorus solubilization and plant growth [53]. Meanwhile, Fusarium, which contains potential plant pathogenic members, showed a relative decrease under CM, suggesting that the improved fungal community structure under mulching may reduce pathogen prevalence and enhance rhizosphere resilience [54]. Enrichment of Tetracladium and Solicoccozyma under residue addition further indicates stimulation of fungal taxa involved in cellulose breakdown and nutrient cycling [55]. Conversely, the CK treatment showed higher abundance of unclassified and oligotrophic fungi, reflecting a less active and possibly more stressed fungal community under nutrient-limited conditions.

#### 3.2.5. Effects of Corncob Returning on Fungal α-Diversity

Corncob returning strongly influenced fungal α-diversity indices in the rhizosphere (Table 3). Compared with CK, corncob incorporation (CI) markedly reduced fungal richness as reflected by ACE and Chao1 indices, whereas corncob mulching (CM) resulted in intermediate values. These changes suggest that residue incorporations may initially favor specific fungal guilds capable of rapidly utilizing corncob substrates, leading to a temporary decline in total richness due to competitive dominance [56]. A similar reduction in fungal richness under lignocellulosic amendment has been reported, associated with the proliferation of specialized saprotrophs and shifts in carbon flow pathways during residue decomposition [57]. In contrast, mulching provided a more gradual nutrient supply and maintained heterogeneous microhabitats, thereby sustaining a more diverse fungal assemblage. Notably, Shannon diversity increased significantly under CI relative to CK, while CM remained comparable to CK. Concurrently, Simpson values were highest under CI and lowest under CK, indicating reduced dominance and enhanced evenness in CI-treated soils. This pattern implies that although CI decreased richness, it promoted a more balanced fungal community structure, likely through stimulation of diverse residue-degrading fungi and suppression of oligotrophic or stress-tolerant taxa [58]. Such restructuring may enhance functional redundancy and resilience in nutrient-enriched environments. The stronger fungal diversification response under CI compared with CM suggests differential assembly mechanisms: incorporation promotes competitive turnover and niche redistribution, whereas mulching supports stable recruitment of saprotrophic and mutualistic taxa. These dynamics align with earlier results showing enrichment of Mortierella and Basidiomycota under organic residue addition, reflecting enhanced lignocellulose degradation capacity and root-associated symbioses [59].

### 3.3. Effects of Corncob Returning on Soil Microbial Biomass C, N, and P

**Figure 6:**
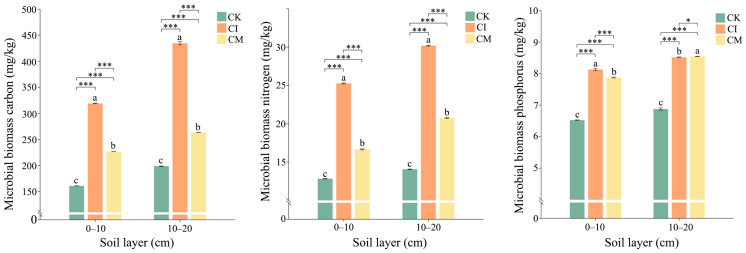
Effects of corncob returning on microbial biomass carbon (MBC), nitrogen (MBN), and phosphorus (MBP) in rhizosphere soil at 0–10 cm and 10–20 cm depths. Different letters indicate significant differences among treatments at *p* < 0.05. Significance levels: * *p* < 0.05, *** *p* < 0.001. Different lowercase letters indicate significant differences among the CK, CI, and CM treatments at the same soil depth (*p* < 0.05), as determined by one-way analysis of variance (ANOVA) followed by Tukey’s honestly significant difference (HSD) post hoc test.

Corncob returning significantly enhanced microbial biomass carbon (MBC), nitrogen (MBN), and phosphorus (MBP) levels in the rhizosphere soil relative to the control (Figure 6). Across both soil layers, corncob incorporation (CI) exhibited the highest microbial biomass, followed by corncob mulching (CM), whereas the no-amendment control (CK) had the lowest values. MBC increased markedly under CI, with values exceeding those in CK by more than two-fold in both the 0–10 cm and 10–20 cm layers. CM also improved MBC, although to a lesser degree. The enhancement in MBC suggests that corncob served as a substantial carbon substrate, promoting microbial growth and turnover [60]. Lignocellulosic residues supply both labile and recalcitrant carbon fractions, enabling rapid microbial colonization and sustained carbon input for microbial metabolism [61]. The superior effect of CI likely arises from improved soil-residue contact and more favorable oxygen conditions for decomposers, accelerating carbon assimilation. MBN followed a similar pattern, with CI inducing the greatest increase relative to CK, particularly in deeper soils (10–20 cm). This is likely reflected in enhanced nitrogen immobilization during active decomposition, whereby microorganisms temporarily sequester nitrogen to support enzymatic activity and biomass synthesis [62]. The pronounced MBN response under CI further indicates that residue incorporation intensifies microbial competition for nitrogen, enhancing N retention within the microbial pool and reducing potential N loss from soil systems [63]. This phenomenon is widely recognized in high-C substrates with elevated C/N ratios, such as corncob biomass [64]. MBP also increased significantly under CI and CM compared with CK, suggesting that microbial uptake and cycling of phosphorus were stimulated by residue amendment. Greater MBP under CI implies enhanced microbial demand for P during biomass formation and enzyme production, consistent with elevated MBC and MBN. Decomposition of lignocellulosic material often triggers microbial P mining, activating phosphatase enzymes and promoting P turnover in soil [65]. The moderate but positive effect of CM highlights the role of mulching in promoting surface P cycling through gradual residue breakdown and fungal-associated P mobilization.

### 3.4. Effects of Corncob Returning on Soil Enzyme Activities

**Figure 7:**
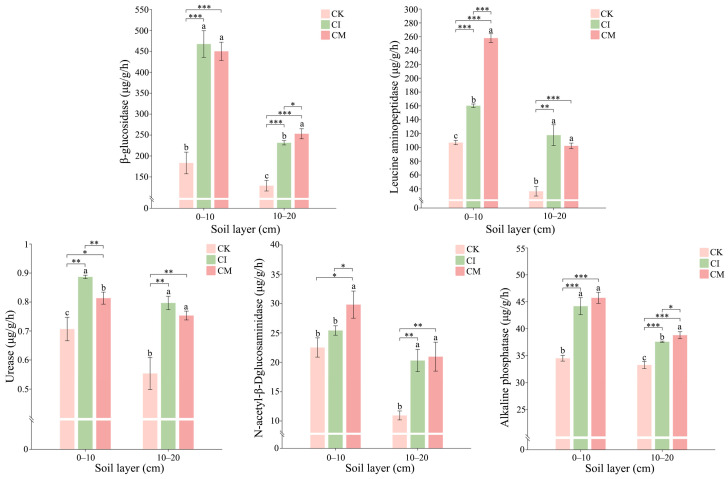
Effects of corncob returning on soil enzyme activities, including β-glucosidase, leucine aminopeptidase, N-acetyl-β-D-glucosaminidase, urease, and alkaline phosphatase, at 0–10 cm and 10–20 cm depths. Different letters indicate significant differences at *p* < 0.05. Significance: * *p* < 0.05, ** *p* < 0.01, *** *p* < 0.001. Different lowercase letters indicate significant differences among the CK, CI, and CM treatments at the same soil depth (*p* < 0.05), as determined by one-way analysis of variance (ANOVA) followed by Tukey’s honestly significant difference (HSD) post hoc test.

Corncob returning markedly enhanced soil enzyme activities associated with carbon, nitrogen, and phosphorus cycling in the rhizosphere (Figure 7).

#### 3.4.1. Carbon-Cycling Enzymes

Activities of β-glucosidase and N-acetyl-β-D-glucosaminidase were significantly elevated under CI and CM relative to CK. β-glucosidase plays a central role in cellulose degradation and reflects labile carbon cycling, while N-acetyl-β-D-glucosaminidase is linked to decomposition of chitin-containing organic compounds. Their increases imply enhanced breakdown of lignocellulosic residues and microbial biomass turnover following corncob addition [66]. The greater stimulation under CI suggests more active microbial colonization and residue decomposition due to improved residue–soil contact and oxygen availability [67].

#### 3.4.2. Nitrogen-Cycling Enzymes

Leucine aminopeptidase and urease showed substantial increases under CI and CM, with urease activity being particularly pronounced in the surface soil under CI. Urease catalyzes urea hydrolysis and is associated with enhanced N mineralization and availability for plant uptake [68]. The strong induction of aminopeptidase suggests intensified protein decomposition and organic N turnover, consistent with elevated microbial biomass nitrogen observed earlier. These responses indicate that corncob amendment stimulates both inorganic and organic N cycling via microbial proteolysis and ammonification [69].

#### 3.4.3. Phosphorus-Cycling Enzymes

Alkaline phosphatase activity increased significantly following CI and CM treatments, with CI showing the highest activity in both soil layers. Phosphatases mediate the release of inorganic P from organic pools, and their activation suggests greater microbial demand for P during residue decomposition and biomass synthesis [70]. The enhanced phosphatase activity under CI aligns with the high microbial biomass P observed previously and demonstrates a strong link between C-rich residue input and stimulated P mobilization pathways [71].

Collectively, these enzyme responses reveal that corncob returning accelerates C-N-P biogeochemical cycling by stimulating key microbial extracellular enzymes. CI produced the strongest stimulation, likely due to efficient substrate incorporation into microbial habitat, while CM provided sustained but comparatively moderate enhancement. The reinforced enzymatic activity corresponds with improved soil nutrient availability and microbial biomass pools, confirming that corncob addition strengthens nutrient transformation networks and promotes a biologically active rhizosphere system [72].

### 3.5. Effects of Corncob Returning on the Growth of E. sessiliflorus

Corncob returning significantly enhanced the growth performance of *E. sessiliflorus* in terms of plant height, stem diameter, leaf area, fruit number, and both aboveground and belowground biomass (Table 4). Both corncob incorporation (CI) and corncob mulching (CM) treatments resulted in significant increases compared to the control (CK), with CM showing the greatest improvements across all growth parameters.

Plant height and stem diameter increased significantly under CI and CM treatments. Plants in the CM treatment reached an average height of 79.67 cm, significantly higher than the CK (55.40 cm). Similarly, stem diameter was also significantly larger in CM (11.40 mm) compared to CK (7.01 mm). These increases in plant height and stem diameter under CI and CM indicate that corncob residue incorporation and mulching enhance structural growth, likely by improving nutrient and water availability in the rhizosphere [73]. Leaf area and fruit number were also significantly enhanced under both residue treatments, with CM showing the highest values. Leaf area in the CM treatment was 170.67 cm^2^ per leaf, compared to 98.41 cm^2^ in the CK. Furthermore, the number of fruits per plant increased from 9.41 fruits in CK to 22.00 fruits in CM, showing a dramatic improvement in reproductive success. These results suggest that corncob residues not only promote vegetative growth but also enhance the plant’s reproductive capacity by improving nutrient uptake and overall plant vigor [74]. Both aboveground and belowground biomass were significantly higher in the CI and CM treatments. Aboveground biomass increased from 103.69 g/plant in CK to 276.93 g/plant in CM, representing a 167% increase. Similarly, belowground biomass also increased, with CM plants exhibiting 220.50 g/plant, compared to 50.27 g/plant in CK. This significant increase in biomass under corncob treatments indicates improved overall plant productivity and suggests that corncob returning enhances both root and shoot growth [75].

### 3.6. Effects of Corncob Returning on Leaf Nutrient Accumulation

**Figure 8:**
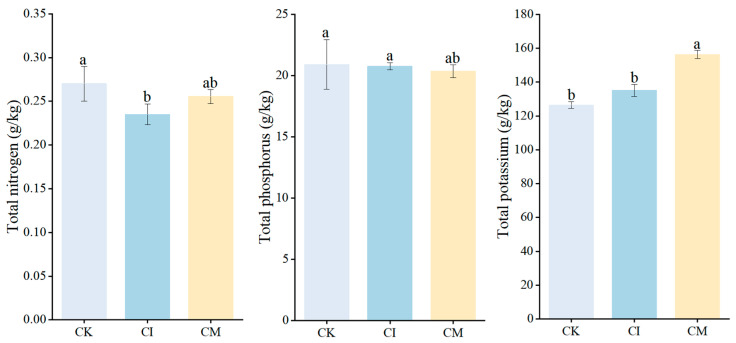
Effects of corncob returning on leaf nitrogen (N), phosphorus (P), and potassium (K) concentrations of *E. sessiliflorus*. Different letters indicate significant differences among treatments at *p* < 0.05.

Corncob returning influenced leaf nutrient status of *E. sessiliflorus*, with distinct responses observed for nitrogen (N), phosphorus (P), and potassium (K) (Figure 8). Leaf N content showed treatment-dependent variation. Compared with CK, corncob incorporation (CI) resulted in a significant decrease in leaf N concentration, whereas corncob mulching (CM) maintained N levels comparable to CK. The reduction under CI likely reflects the temporary microbial N immobilization associated with decomposition of high C/N residues, which can reduce plant-available N during early decomposition phases [76]. Conversely, CM moderated this effect, likely due to gradual N mineralization and improved surface moisture conditions that sustained N availability. Similar patterns have been reported in organic-amended soils, where microbial competition for N can initially limit plant uptake until mineralization progresses [77]. Leaf P concentration increased under both CI and CM relative to CK, though differences were not statistically significant. This trend aligns with the observed increase in phosphatase activity and available soil P in residue-treated soils, indicating enhanced P mobilization and uptake. The improvement may result from increased microbial turnover, organic acid secretion, and enhanced mycorrhizal colonization following residue application [78]. Organic residue inputs often stimulate P-solubilizing microorganisms and improve soil P bioavailability, thereby supporting P acquisition by perennial medicinal plants [79]. Leaf K content increased substantially under CM, while CI exhibited intermediate levels and CK remained lowest. The strong K enrichment under mulching suggests that surface residue placement facilitated rapid K release due to leaching and microbial processing of corncob material, which is known to contain readily exchangeable potassium fractions [80]. Enhanced soil moisture and hyphal proliferation under CM further contributed to improved K mobility and uptake [81]. Potassium plays a critical role in osmotic regulation, photosynthetic efficiency, and fruit development, and its increase under CM supports the improved plant growth and reproductive performance observed earlier.

### 3.7. Redundancy Analysis of Soil Properties, Enzyme Activity, Plant Traits and Microbial Communities

**Figure 9:**
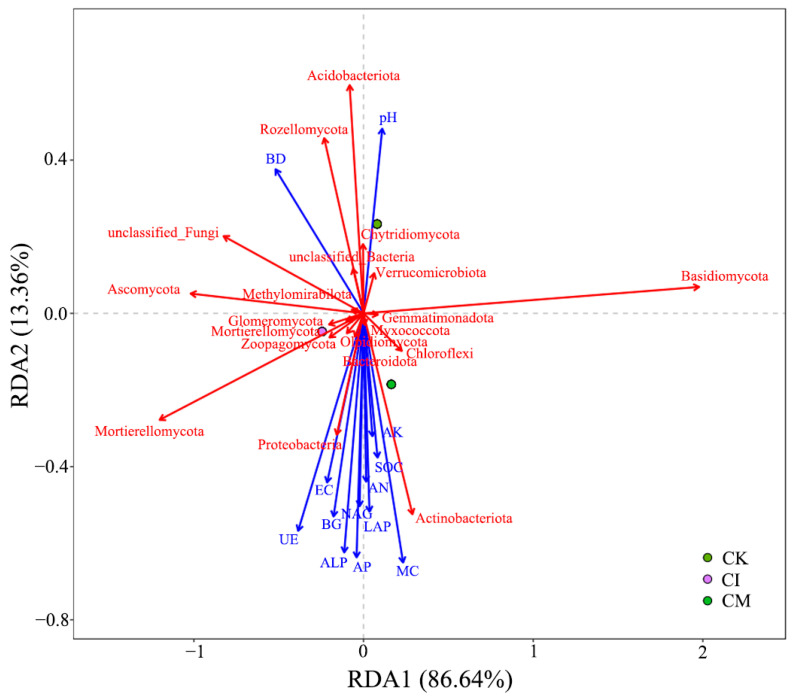
Redundancy analysis (RDA) of soil physicochemical properties, enzyme activities, and microbial community composition under different corncob returning treatments. BD, bulk density; pH, potential of hydrogen; EC, electrical conductivity; MC, moisture; SOC, soil organic carbon; UE, urease; BG, β-glucosidase; ALP, alkaline phosphatase; LAP, leucine aminopeptidase; NAG, N-acetyl-β-D-glucosaminidase; AP, available phosphorus; AN, available nitrogen; AK, available potassium. The red vectors represent the microbial community composition, while the blue vectors correspond to the soil physicochemical properties and enzyme activities.

The RDA ordination revealed clear associations between soil environmental variables and microbial community structure following corncob returning (Figure 9). The first RDA axis explained 86.64% of the variation, indicating that soil physicochemical properties and enzyme activities strongly influenced microbial assembly patterns. Treatments with corncob amendments, especially mulching, were separated from the control, suggesting distinct shifts in microbial composition under residue addition.

Among environmental variables, soil organic carbon (SOC) and microbial biomass carbon (MBC) were strongly positively correlated with Proteobacteriota and Bacteroidota, phyla typically regarded as copiotrophs stimulated by readily available carbon substrates [82]. Enhanced β-glucosidase (BG), urease (UE), and alkaline phosphatase (ALP) activities were also positively associated with these taxa, implying their role in accelerating C-, N-, and P-turnover under corncob amendment [83].

Conversely, Acidobacteriota and Chloroflexi were associated with CK plots and negatively correlated with nutrient-related variables, reflecting preference for nutrient-limited conditions [84]. Increased soil moisture (MC) and reduced bulk density (BD) under mulching aligned with fungal phyla such as Basidiomycota and Mortierellomycota, supporting earlier findings of enhanced fungal activity in organic mulched systems [85].

**Figure 10:**
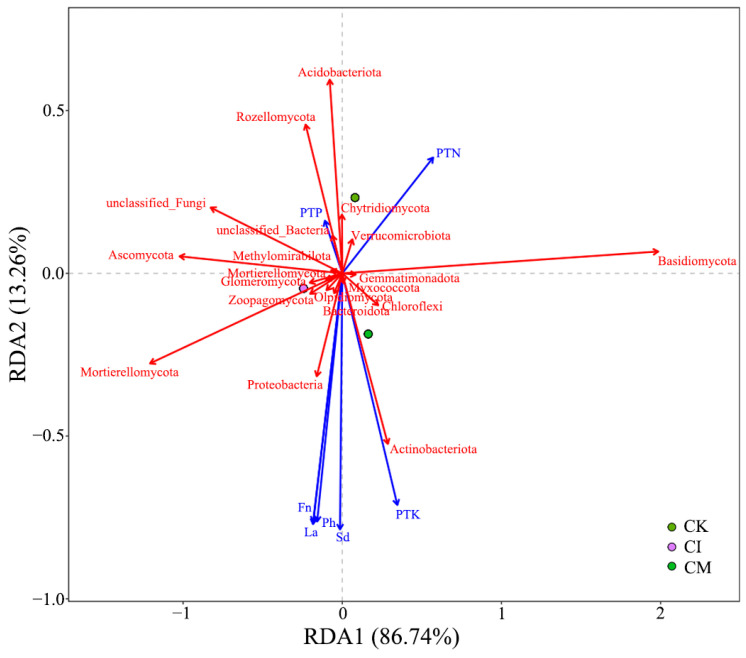
RDA of plant traits and microbial community composition following corncob returning. Ph, plant height; La, leaf area; Fn, fruit number; Sd, stem diameter; PTN, plant total nitrogen; PTP, plant total phosphorus; PTK, plant total potassium. The red vectors represent the microbial community composition, while the blue vectors represent the plant traits.

The RDA linking plant traits and microbial communities (Figure 10) further demonstrated strong plant–soil–microbe interactions. The first axis explained 88.74% of the variation, indicating a dominant role of residue-driven plant–microbiome feedback. Plant nutrient concentrations (PTN, PTP, PTK), plant height (Ph), leaf area (La), and fruit number (Fn) were strongly correlated with Basidiomycota, Mortierellomycota, and copiotrophic bacterial taxa. These microbial groups are known to enhance nutrient solubilization, produce growth-promoting metabolites, and improve rhizosphere nutrient turnover, collectively supporting host plant performance [86].

In contrast, CK samples clustered near oligotrophic taxa such as Acidobacteriota, suggesting that nutrient-poor conditions limited plant growth and favored stress-adapted microbial consortia. These patterns align with our earlier results showing improved nutrient uptake, especially P and K, and enhanced biomass accumulation under residue returning treatments.

### 3.8. Correlation Analysis Between Soil Properties, Microbial Functions, and Plant Performance

**Figure 11:**
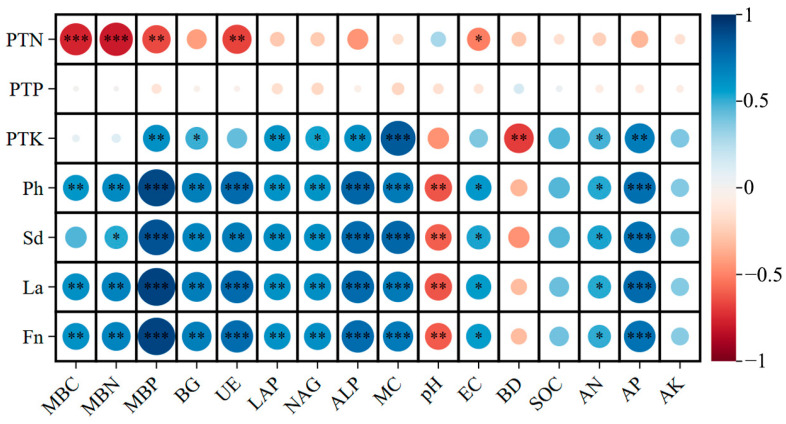
Correlation heatmap between soil physicochemical properties, microbial indicators, and plant traits under different corncob returning treatments. MBC, microbial biomass carbon; MBN, microbial biomass nitrogen; MBP, microbial biomass phosphorus; BG, β-glucosidase; LAP, leucine aminopeptidase; NAG, N-acetyl-β-D-glucosaminidase; UE, urease; ALP, alkaline phosphatase; MC, soil moisture; BD, bulk density; SOC, soil organic carbon; AN, available nitrogen; AP, available phosphorus; AK, available potassium; Ph, plant height; Sd, stem diameter; La, leaf area; Fn, fruit number; PTN, plant total nitrogen; PTP, plant total phosphorus; PTK, plant total potassium. Significance levels: * *p* < 0.05, ** *p* < 0.01, *** *p* < 0.001.

The correlation heatmap revealed strong and coordinated interactions among soil biochemical attributes, microbial activity, and plant growth parameters following corncob returning (Figure 11). Overall, soil microbial biomass, enzyme activities, and nutrient availability exhibited significant positive correlations with plant nutrient status and growth traits, underscoring the central role of rhizosphere biochemical processes in mediating plant performance under residue amendment.

Microbial biomass indicators (MBC, MBN, MBP) showed strong positive correlations with plant total nitrogen (PTN), phosphorus (PTP), and potassium (PTK), as well as plant height (Ph), stem diameter (Sd), leaf area (La), and fruit number (Fn). These relationships indicate enhanced microbial nutrient immobilization and turnover improved nutrient supply to *E. sessiliflorus* [87]. Microbial biomass is widely recognized as a sensitive indicator of soil fertility and an active reservoir for nutrient release, particularly under organic amendments [88], consistent with the strong plant–microbial coupling observed here.

Carbon-, nitrogen-, and phosphorus-cycle enzymes (BG, LAP, NAG, UE, ALP) were positively associated with plant growth and foliar nutrient content. This suggests that enzyme-mediated nutrient mineralization supported nutrient uptake and growth, particularly for N and P. Such enzyme–plant linkages are well documented in residue-amended agricultural soils, where microbial enzymes accelerate nutrient cycling and enhance root access to organic nutrient pools [89]. The stronger correlations of urease (UE) and ALP with plant traits highlight their role in supporting nitrogen mineralization and phosphorus mobilization under corncob amendment.

Soil moisture (MC), organic carbon (SOC), and available nutrients (AN, AP, AK) exhibited strong positive correlations with plant traits and leaf nutrient contents. Conversely, soil bulk density (BD) showed negative correlations, reflecting the beneficial role of residue-induced structural improvement on plant growth. Increased SOC and moisture create favorable conditions for microbial proliferation and nutrient release, reinforcing the cascading effect of soil carbon enrichment on plant productivity [90].

### 3.9. Integrated Correlations Among Soil Properties, Microbial Communities, Enzyme Activities, and Plant Traits

**Figure 12:**
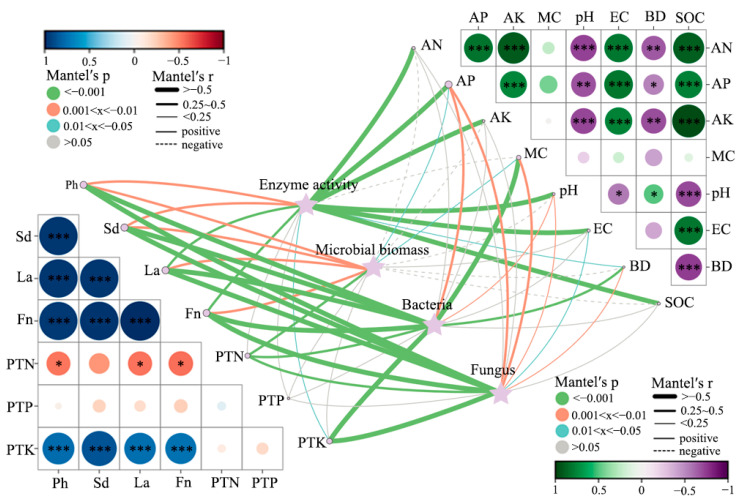
Integrated correlation network shows relationships among soil physicochemical properties, microbial biomass, enzyme activities, microbial communities, and plant traits under corncob returning treatments. Bubble colors represent correlation coefficients; bubble size corresponds to *p*-values. Line thickness and color indicate correlation direction and strength. Significance levels: * *p* < 0.05, ** *p* < 0.01, *** *p* < 0.001.

The interactive correlation network (Figure 12) provides a holistic view of the relationships among soil physicochemical variables, microbial parameters, enzyme activities, and plant performance after corncob returning. Microbial biomass showed strong positive associations with key C–N–P cycling enzymes (β-glucosidase, leucine aminopeptidase, N-acetyl-β-D-glucosaminidase, urease, and alkaline phosphatase). These correlations indicate that microbial proliferation and enzyme induction co-occur under corncob amendment, promoting nutrient mineralization and rhizosphere nutrient turnover [91]. Such relationships are consistent with residue-driven increases in carbon substrates and microbial metabolic activity, enhancing extracellular enzyme production [92]. Both bacteria and fungi were positively correlated with plant physiological indicators including height, stem diameter, leaf area, and fruit number. Fungal communities showed particularly strong associations with plant nutrient status (PTN, PTP, PTK), highlighting their role in mobilizing complex organic nutrients and enhancing nutrient uptake in perennial root systems [93]. These patterns support the view that fungi serve as key mediators in residue-induced nutrient cycling and plant performance, particularly under conditions where lignocellulosic inputs are abundant [94].

Soil organic carbon (SOC), moisture (MC), and available nutrients (AN, AP, AK) were closely linked with microbial activity and plant traits, emphasizing carbon enrichment and water balance as primary drivers of rhizosphere functioning. In contrast, bulk density (BD) and pH exhibited negative or weak correlations with biological variables, suggesting that physical structure improvements and slight pH reduction indirectly facilitated microbial and plant responses. The strong positive interactions between SOC, microbial biomass, and plant productivity reinforce the concept of soil C priming and nutrient cycling enhancement following organic residue returning [95]. Additionally, the central role of enzyme activity in connecting soil properties with plant responses highlights enzymatic nutrient mobilization as a mechanistic bridge in the soil–plant continuum.

### 3.10. Partial Least Squares Structural Equation Modeling (PLS-SEM) of Soil–Microbe–Plant Interactions

**Figure 13:**
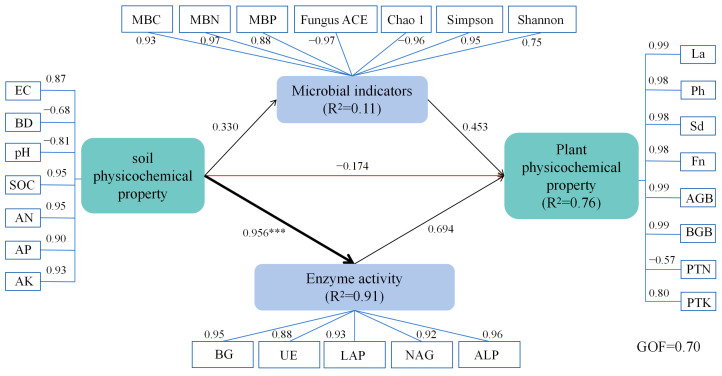
The partial least squares structural equation modeling (PLS-SEM) revealed the direct and indirect effects of soil physicochemical properties, microbial indicators, and enzyme activities on plant traits under maize corncob returning conditions. The arrow colors denote positive (black) or negative (red) path directions, with numbers along the arrows indicating path coefficients. Line widths reflect the magnitude of the effects. Asterisks denote significance levels (*** *p* < 0.001). R^2^ values represent the proportion of explained variance for each latent variable. A goodness-of-fit (GOF) value of 0.70 indicates a satisfactory model fit.

The PLS-SEM analysis (Figure 13) provides a comprehensive understanding of the relationships between soil physicochemical properties, microbial indicators, enzyme activity, and plant performance under corncob returning treatments. The model fit was strong, with a goodness of fit (GOF) value of 0.70, indicating that the model explains a significant portion of the variance in soil–microbe–plant interactions.

To further validate the PLS-SEM model’s reliability, additional goodness-of-fit (GOF) indices and robustness tests were supplemented in line with standard PLS-SEM reporting guidelines. First, the standardized root mean square residual (SRMR)—a key index for assessing model fit in PLS-SEM—was calculated as 0.042, which is well below the threshold of 0.08, indicating excellent agreement between the observed data and the hypothesized model. Second, the R^2^ values of latent variables were detailed to reflect the model’s explanatory power: enzyme activity (R^2^ = 0.91), microbial indicators (R^2^ = 0.11), and plant physicochemical property (R^2^ = 0.76). Among these, the high R^2^ values for enzyme activity (0.91) confirm that the model effectively explains the variance in these core variables, while the moderate R^2^ for plant physicochemical property (0.76) aligns with the complex, multi-factorial nature of plant growth responses.

For robustness verification, a bootstrapping test with 1000 resamples was conducted to assess the statistical significance of path coefficients. The results showed that all key path coefficients (e.g., soil physicochemical properties → Enzyme activity: path = 0.956, *p* < 0.001; enzyme activity → plant physicochemical property: path = 0.694, *p* < 0.001) had bootstrap confidence intervals (95%) that did not include zero, confirming the stability and reliability of the hypothesized causal relationships. No multicollinearity issues were detected among latent variables (variance inflation factor, VIF < 3.0), further supporting the model’s structural validity [96].

Soil physicochemical properties play a key regulatory role in influencing microbial indicators, enzyme activity, and plant growth. In this study, soil physicochemical properties (characterized by electrical conductivity (EC), bulk density (BD), pH, soil organic carbon (SOC), available nitrogen (AN), available phosphorus (AP), and available potassium (AK)) exerted a positive direct effect on microbial indicators (encompassing microbial biomass carbon (MBC), microbial biomass nitrogen (MBN), microbial biomass phosphorus (MBP), and fungal diversity indices; path coefficient = 0.330), while simultaneously exhibiting a strong and significant positive direct effect on enzyme activity (path coefficient = 0.956, *p* < 0.001). Enzyme activity, represented by high-loading hydrolytic enzymes (β-glucosidase (BG), urease (UE), leucine aminopeptidase (LAP), N-acetyl-β-D-glucosaminidase (NAG), and alkaline phosphatase (ALP); factor loadings = 0.88–0.96), explained 91% of the variance (R^2^ = 0.91). In contrast, soil physicochemical properties showed a weak negative direct effect on plant physicochemical properties (path coefficient = −0.174), reflecting transient salt stress induced by a slight increase in EC (loading = 0.87) during the initial stage of maize corncob decomposition. However, this adverse effect was offset by the indirect positive effects of biotic factors. Notably, SOC, AN, AP, and AK contributed strongly to the soil physicochemical property latent variable (loadings = 0.90–0.95), whereas EC and pH showed negative associations (loadings = −0.68 to −0.87). This indicates that soil physicochemical properties shape microbial biomass and diversity through pH and nutrient availability, and also serve as important substrates for enzyme synthesis. In particular, SOC input from maize corncobs (loading = 0.95) fuels the catalytic activity of carbon and nitrogen cycling enzymes, thereby linking soil abiotic conditions to belowground biological functions [97]. Furthermore, soil enzyme activity had a significant impact on plant physicochemical properties (e.g., plant growth and root development) (path coefficient = 0.694), influencing the plant’s ability to absorb essential nutrients such as nitrogen, phosphorus, and potassium. This impact occurs through both direct and indirect pathways, potentially involving improved soil structure, enhanced nutrient availability, and optimized microbial communities. Changes in soil physicochemical properties not only affect the composition of microbial communities but also alter the growing environment for plants, thereby influencing their physiological functions and development. Thus, the influence of soil physicochemical properties on ecosystem functions is multifaceted, affecting both the structure and function of microbial communities, as well as regulating nutrient availability for plants, which in turn impacts plant growth and development [98].

Enzyme activity, regulated by soil conditions, subsequently plays a central role in modulating plant physicochemical properties (e.g., plant height (Ph), stem diameter (Sd), leaf area (La), fruit number (Fn), aboveground biomass (AGB), and belowground biomass (BGB)), with a strong positive direct effect (path coefficient = 0.694). This effect, combined with the positive contribution of microbial indicators (path coefficient = 0.453), collectively accounted for 76% of the variance in plant traits (R^2^ = 0.76), where most plant parameters (e.g., AGB, BGB, Ph, Sd) exhibited high positive factor loadings (0.98–0.99). This suggests that enzyme-mediated nutrient release is crucial for improving plant nutrient uptake and biomass production. These findings are consistent with previous studies indicating that soil enzymes facilitate the release of limiting nutrients, such as nitrogen, phosphorus, and potassium, thereby promoting plant growth [99]. The strong path coefficient from enzyme activity to plant traits (path = 0.694) emphasizes the core role of enzymatic processes in driving plant productivity in corncob-amended soils, where enzyme-mediated nutrient release directly supports plant growth and biomass accumulation.

Microbial indicators, including microbial biomass carbon (MBC), microbial biomass nitrogen (MBN), microbial biomass phosphorus (MBP), and fungal diversity (measured by Fungus ACE, Chao1, and Simpson indices), were influenced by soil physicochemical properties (path = 0.330). Microbial indicators exerted a positive direct effect on plant traits (path coefficient = 0.453), but this effect was weaker than that of enzyme activity. This supports the notion that microbial functional activity, rather than mere richness or diversity, is the dominant driver of plant growth in maize corncob-returned soils. Notably, plant total nitrogen (PTN) showed a negative loading (−0.57) on the plant physicochemical property latent variable, which is consistent with the “nitrogen immobilization” phenomenon: during the initial decomposition of maize corncobs, microbes compete with plants for available nitrogen, temporarily limiting plant nitrogen uptake [100]. This observation suggests that complementary nitrogen inputs may mitigate such transient nutrient limitation and optimize plant performance in maize corncob-returned systems.

### 3.11. Challenges and Prospects of Corncob Residue Application

The high carbon-to-nitrogen (C/N) ratio of corncob residues (approximately 50:1) can induce short-term nitrogen immobilization during the early decomposition stage. When corncobs are incorporated into soil, soil microorganisms prioritize utilizing available soil mineral nitrogen to decompose the lignocellulosic carbon in corncobs, leading to temporary nitrogen deficiency for crop uptake [101]. This phenomenon may inhibit the growth of *E*. *sessiliflorus* in the first growing season post-application. To address this, pre-treatment measures such as crushing corncobs to 2–5 mm (to accelerate decomposition) or co-composting with nitrogen-rich materials (e.g., livestock manure, urea) can adjust the C/N ratio to 25–30:1, minimizing nitrogen immobilization while maintaining carbon input [102].

Unsterilized corncob residues may harbor pathogenic propagules (e.g., Fusarium oxysporum, Rhizoctonia solani), posing a risk of soil-borne pathogen proliferation in continuous cropping systems [103]. Corncob residues with high moisture retention (especially under mulching) can create a favorable microhabitat for pathogen growth, potentially increasing disease incidence in *E. sessiliflorus.* Mitigation strategies include solarization of corncobs (exposing to 60–70 °C for 72 h) to inactivate pathogens before application, or rotating *E. sessiliflorus* with disease-resistant crops (e.g., Triticum aestivum cv. ‘Kangmai 18’) to break pathogen life cycles [104].

If corncob raw materials are sourced from fields contaminated with heavy metals (e.g., Cd, Pb, As), returning them to soil may lead to heavy metal accumulation over long-term application [105]. Corncobs can adsorb heavy metals from the growing environment, and continuous incorporation may increase their bioavailability in soil, posing risks to plant safety and soil health. To prevent this, it is recommended to screen corncob raw materials using inductively coupled plasma mass spectrometry (ICP-MS) to ensure heavy metal concentrations meet national soil quality standards (GB 15618-2018, China) [106]. For corncobs with mild heavy metal contamination, pre-treatment with 0.1 mol/L citric acid can reduce heavy metal bioavailability by 30–40% before soil application [107].

These challenges do not negate the environmental and agronomic benefits of corncob returning but highlight the need for context-specific management to maximize its sustainability in *E. sessiliflorus* cultivation systems.

## 4. Conclusions

This study demonstrates that corncob residue recycling is an effective strategy for improving soil quality, microbial function, and plant productivity in *E. sessiliflorus* cultivation systems in cold-temperate regions. This study was strictly centered on the three hypotheses and corresponding objectives proposed in the Introduction, and all key findings directly respond to these research premises. Specifically, our results fully supported Hypothesis (i) compared to the unamended control, corncob returning significantly enhanced soil organic carbon, moisture content, and the availability of key nutrients (N, P, and K), while reducing soil bulk density, thus creating a more favorable rhizosphere environment for plant growth. Both corncob incorporation (CI) and corncob mulching (CM) treatments significantly increased microbial biomass and nutrient-cycling enzyme activities—particularly β-glucosidase, urease, and alkaline phosphatase—which in turn accelerated soil C–N–P turnover. For Hypothesis (ii), we confirmed that corncob amendments reshaped rhizosphere microbial communities—enriching copiotrophic bacteria (e.g., Proteobacteria) and lignocellulose-degrading fungi (e.g., Basidiomycota)—while microbial functional activation (rather than diversity) dominated nutrient cycling, fulfilling the objective of characterizing microbial community shifts and functional potential. Regarding Hypothesis (iii), these biochemical and microbial improvements directly translated into enhanced plant nutrient accumulation and substantial increases in biomass production, with plant height increasing by 44% and fruit number by 136%.

The PLS-SEM analysis in this study demonstrates that the impact of soil physicochemical properties on plant growth is primarily mediated through enzyme activity and microbial activity. Soil improvements enhance microbial environments and enzyme activity, increasing nutrient availability and supporting plant growth. Additionally, microbial functional activity (rather than microbial richness) plays a more significant role in plant growth promotion. This highlights the multi-faceted effects of soil health on agricultural ecosystem functions, suggesting that improving soil physicochemical properties and microbial activity can effectively promote healthy plant growth.

Overall, the findings emphasize a clear mechanistic pathway: corncob returning → enhanced soil physicochemical properties → microbial indicators activation and enzymatic activity → increased nutrient availability → improved plant productivity. Corncob recycling provides a sustainable and scalable approach for improving soil fertility and crop performance, particularly in perennial medicinal plant systems. This study strongly supports the use of corncob mulching and incorporation as effective ecological practices in cold-region agroecosystems, offering a viable solution for utilizing agricultural residues to promote sustainable farming and functional plant cultivation. While the two-year trial in this study has effectively captured the short-to-medium-term effects of corncob returning on the soil–plant-microbe system, it should be noted that the decomposition of lignocellulosic corncob residues is a long-term ecological process. Therefore, in future research, we will extend the monitoring period to further clarify the long-term dynamics of soil carbon sequestration and microbial community succession induced by corncob decomposition, thereby verifying the sustainability of this practice.

## Figures and Tables

**Table 1:** Effects of corncob returning on soil pH, electrical conductivity, moisture content, bulk density, and soil organic carbon in *E. sessiliflorus* rhizosphere (*n* = 3).

Soil Layers	Treatment	pH	Conductivity /(mS/cm)	Moisture Content /(%)	Bulk Density /(g/cm^3^)	SOC /(g/kg)
0–10 cm	CK	8.59 ± 0.03 a	0.11 ± 0.02 b	14.98 ± 0.02 c	1.23 ± 0.03 b	16.25 ± 0.90 c
	CI	8.35 ± 0.03 ab	0.19 ± 0.01 a	18.34 ± 0.03 b	1.28 ± 0.01 a	18.18 ± 0.33 b
	CM	8.12 ± 0.02 c	0.19 ± 0.01 a	25.37 ± 0.06 a	1.12 ± 0.02 c	20.47 ± 0.19 a
10–20 cm	CK	9.24 ± 0.60 a	0.12 ± 0.01 b	20.13 ± 0.04 c	1.31 ± 0.03 a	14.34 ± 0.87 a
	CI	8.54 ± 0.02 b	0.14 ± 0.01 a	23.04 ± 0.02 b	1.30 ± 0.01 a	14.90 ± 0.44 a
	CM	8.64 ± 0.04 b	0.13 ± 0.01 b	26.51 ± 0.20 a	1.23 ± 0.01 b	15.32 ± 0.10 a

Note: Different letters within the same soil layer indicate significant differences at *p* < 0.05.

**Table 2:** α-diversity indices of soil bacterial communities under different corncob returning treatments (*n* = 3).

Sample ID	ACE	Chao1	Simpson	Shannon
CK	1287 ± 15.79 b	1283 ± 16.93 b	0.999 ± 0.01 a	9.660 ± 0.10 a
CI	1298 ± 8.26 ab	1297 ± 9.49 ab	0.997 ± 0.01 a	9.586 ± 0.09 a
CM	1305 ± 8.97 a	1329 ± 49.20 a	0.996 ± 0.02 a	9.554 ± 0.03 a

Note: Different letters indicate significant differences among treatments at *p* < 0.05.

**Table 3:** α-diversity indices of soil fungal communities under different corncob returning treatments (*n* = 3).

Sample ID	ACE	Chao1	Simpson	Shannon
CK	529 ± 9.26 ab	537 ± 12.41 a	0.873 ± 0.01 c	5.279 ± 0.08 b
CI	453 ± 10.53 bc	454 ± 19.75 b	0.975 ± 0.00 a	6.655 ± 0.21 a
CM	490 ± 15.43 b	494 ± 16.36 ab	0.906 ± 0.00 b	5.054 ± 0.10 b

Note: Different letters indicate significant differences at *p* < 0.05.

**Table 4:** Effects of corncob returning on aboveground growth traits of *E. sessiliflorus* (*n* = 3).

Treatments	Plant Height /(cm)	Stem Diameter /(mm)	Leaf Area /(cm^2^/Leaf)	Fruit Number /(Number/Plant)	Aboveground Biomass /(g/Plant)	Belowground Biomass /(g/Plant)
CK	55.40 ± 3.19 c	7.01 ± 0.24 c	98.41 ± 5.09 b	9.41 ± 1.49 b	103.69 ± 3.07 c	50.27 ± 1.26 c
CI	76.33 ± 1.53 b	10.00 ± 0.53 b	163.00 ± 6.56 a	20.67 ± 1.53 a	238.53 ± 5.42 b	182.83 ± 3.49 b
CM	79.67 ± 4.04 ab	11.40 ± 0.46 a	170.67 ± 2.08 a	22.00 ± 1.00 a	276.93 ± 11.85 a	220.50 ± 5.22 a

Note: Different letters indicate significant differences at *p* < 0.05.

## Data Availability

The original contributions presented in this study are included in the article. Further inquiries can be directed to the corresponding author.
